# Impact of demographic factors on chronic pain among adults in the United States

**DOI:** 10.1097/PR9.0000000000001009

**Published:** 2022-06-06

**Authors:** Peter M. Mullins, Robert J. Yong, Neil Bhattacharyya

**Affiliations:** Departments of a Emergency Medicine and; b Anesthesiology, Perioperative, and Pain Medicine, Brigham and Women's Hospital, Harvard University, Boston, MA, USA; c Department of Otolaryngology, Massachusetts Eye and Ear & Harvard Medical School, Boston, MA, USA

**Keywords:** Demographics, Chronic pain, National health interview survey

## Abstract

**Introduction:**

Chronic pain affects more than 1 in 5 American adults, and its effects are not evenly distributed throughout the population.

**Methods:**

Using the National Health Interview Survey (NHIS), a household-based annual survey of self-reported health status of U.S. adults, this cross-sectional study describes differences in the prevalence of chronic pain and its effects across socioeconomic groups.

**Results:**

In univariate analyses, chronic pain was more prevalent among female respondents, persons with lower educational attainment, non-Hispanic White individuals, and those who were insured as well as those who were married. After accounting for all other demographic factors, age, female sex, and lower educational attainment were associated with higher odds of having chronic pain, whereas Hispanic and non-Hispanic Black race were associated with lower odds. Despite lower odds of having chronic pain, Hispanic and non-Hispanic Black race were associated with greater odds of reporting more severe pain than White race. There were no significant differences across race in the effects of pain on life, work, or family, although female sex and lower educational attainment were associated with greater effects of pain on these domains. Educational attainment was the only characteristic associated with greater odds of ineffective pain management after accounting for all other demographic factors.

**Conclusions:**

Implications for reducing disparities in the treatment of chronic pain are discussed.

## Abstract 2

Using the 2019 National Health Interview Survey, this study investigates associations between demographic factors and chronic pain among adults in the United States.

## 1. Introduction

Chronic pain affects a significant portion of the U.S. population, with recent data estimating more than 1 in 5 American adults experiences chronic pain.^23^ Chronic pain is commonly defined as pain persisting beyond normal tissue healing time and is typically regarded as pain that lasts beyond 3 months.^21^ Chronic pain represents a considerable burden to the U.S. health care system because estimates in 2010 ranged up to $635 billion in health care costs annually; presently, this figure is likely an underestimate.^7^ In addition, chronic pain affects economic productivity with a resulting estimated $79.9 billion in lost wages in 2019.^23^ Chronic pain is thus an area of importance to public health and medicine in the United States.

There has been increasing interest in differential effects of chronic pain across social groups. Disparities, or the unequal treatment of patients on the basis of race, ethnicity, or other demographic characteristics, exist within both the prevalence of chronic pain as well as its treatment.^10^ Previous studies of treatment-seeking populations have demonstrated that chronic pain is more common among older patients as well as among those of lower socioeconomic status and lower educational attainment.^3^ Data from the year 2000 suggest non-Hispanic Black adults and Hispanic adults are more likely than non-Hispanic White adults to experience severe chronic pain, although there have been few more recent household-based population studies of this question.^18^ Previous studies have found increasing socioeconomic disparities in pain from 2002 through 2018.^24^ An updated investigation of the most contemporaneous available data into disparities in chronic pain would therefore have important public health implications.

In this study, we investigate demographics and disparities in chronic pain among adults in the United States using the National Health Interview Survey (NHIS). National Health Interview Survey, a household-based survey of self-reported health status of U.S. adults conducted annually by the National Center for Health Statistics (NCHS), has been widely used to determine the prevalence of a number of health conditions. We have recently quantified the prevalence and relative impact of chronic pain in the United States using this data set.^23^ Using a module included in the 2019 edition of NHIS regarding chronic pain, we calculate national-level estimates of the prevalence of chronic pain over multiple demographic categories, including race, ethnicity, level of education, and insurance status.

## 2. Methods

This study involved a cross-sectional analysis using the NHIS for calendar year 2019 as conducted by the NCHS in the United States. National Health Interview Survey is a nationally representative survey conducted annually since 1957 that investigate illness and disability and the use of health services because of these conditions. National Health Interview Survey is a cross-sectional household interview survey targeting the civilian noninstitutionalized population of the United States. National Health Interview Survey is conducted using a face-to-face interview format using geographically clustered sampling techniques. National Health Interview Survey data are weighted such that they can provide nationally representative estimates using these sampling weights. As part of the 2019 redesign of the NHIS survey, a special component with data fields for acute and chronic pain were included in this national survey. In 2019, a series of questions regarding acute and chronic pain were added to the survey asking respondents to report their experiences of pain over the previous 3 months. This study was reviewed by Partners Healthcare Committee on Clinical Investigations and was deemed exempt from institutional review board review because it uses a publicly available, deidentified data set. The NHIS data set was restricted to adult patients (age 18 years or older), and standard demographic data were extracted. Then, from the chronic pain module, data fields were extracted and processed.

Individuals reporting chronic pain were determined based on the reported frequency of pain over the past 3 months as previously described.^23^ Individuals were separated into those with and without chronic pain. First, demographic factors associated with those individuals reporting chronic pain were tabulated with univariate analysis. Demographic factors examined included age, sex, education level (less than high school, high school graduate, college or associate degree, or advanced or professional degree), race (grouped as Hispanic, non-Hispanic White, non-Hispanic Black, or other), insurance status, and marital status (married or unmarried).

The data set was then restricted to those individuals reporting chronic pain, and univariate associations between demographic factors and measures of pain impact were studied. Chronic pain–related outcome variables examined included amount of pain experienced, how often pain limited life or work (most days or every day vs never or some days), how often pain affected family life (most days or every day vs never or some days), and pain management effectiveness (little or not effective vs very effective or somewhat effective, as defined by the respondent). Data are reported as the population estimate or population percentage with 95% confidence intervals. Chi-square analysis with significance set at *P* = 0.05 was used for univariate statistical comparisons. Missing data for responses were excluded from subsequent analysis.

Next, for each chronic pain outcome variable above, multivariate analysis using dichotomous logistic regression analysis was performed including all demographic factors in the model as independent variables, with all demographic variables entered simultaneously (Table 1). Multivariate associations for the presence or absence of chronic pain and the subsequent outcome measures for chronic pain adjusting for other demographic factors were determined. Data are reported as odds ratios with 95% confidence intervals. Statistical significance was once again set at *P* = 0.05. All analyses were conducted using IBM SPSS for (Windows), version 25.0 (IBM Corp, Armonk, NY).

**Table 1:** Distribution of chronic pain prevalence according to demographic factors.

Variable	% With chronic pain	95% CI	% Without chronic pain	95% CI	*P*
Sex					
Male	19.1	18.3–20.0	80.9	80.0–81.7	<0.001
Female	21.8	21.0–22.7	78.2	77.3–79.0	
Education level					
Less than high school	27.5	25.9–29.5	72.3	70.5–74.1	<0.001
High school graduate	21.7	20.8–22.6	78.3	77.4–79.2	
College or associate degree	18.1	17.2–19.1	81.9	80.9–82.8	
Advanced or professional degree	13.2	12.1–14.3	86.8	85.7–87.9	
Race					
Hispanic	13.1	12.0–14.3	86.9	85.7–88.0	<0.001
Non-Hispanic White	23.7	22.9–24.4	76.3	75.6–77.1	
Non-Hispanic Black	19.4	18.0–21.0	80.6	79.0–82.0	
Other	12.8	11.3–14.6	87.2	85.4–88.7	
Insurance status					
Insured	20.8	20.2–21.5	79.2	78.5–79.8	0.005
Uninsured	17.8	15.9–19.8	82.2	80.2–84.1	
Marital status					
Married	20.0	19.2–20.7	80.0	79.3–80.8	0.013
Not married	21.3	20.4–22.2	78.7	77.8–79.6	

## 3. Results

The 2019 NHIS included data from 31,997 adult respondents, representing an estimated 244.6 million U.S. adults (95% CI: 237.7–251.4 million). The household response rate for NHIS in 2019 was 61.1%. Among U.S. adults, 50.2 million (48.2–52.2 million) reported chronic pain. There were significant differences in the prevalence of chronic pain across sex, with 21.8% (21.0–22.7) of female respondents reporting chronic pain compared with 19.1% (18.3–20.0) of male respondents (*P* < 0.001). Higher educational attainment was associated with lower rates of chronic pain because 27.5% (25.9–29.5) of those reporting less than high school education endorsing chronic pain compared with 13.2% (12.1–14.3) of advanced or professional degree holders (*P* < 0.001). Across racial groups, non-Hispanic White adults had the highest reported rates of chronic pain at 23.7% (22.9–24.4). Uninsured respondents reported lower rates of chronic pain at 17.8% (15.9–19.8) compared with 20.8% (20.2–21.5) of insured adults. Married respondents endorsed significantly lower rates of chronic pain at 20.0% (19.2–20.7) vs 21.3% (20.4–22.2) of unmarried adults (Table 1).

Table 2 presents multivariate analysis of demographic factors associated with the presence of chronic pain among U.S. adults. Each of the demographic factors was independently associated with the presence of chronic pain except for insurance status, which was not. Notably, increased age, female sex, decreasing level of education, and nonmarried status were associated with higher likelihood of the presence of chronic pain. Hispanic, non-Hispanic Black, and other racial groups had lower reported prevalence of chronic pain on multivariate analysis.

**Table 2:** Multivariate analysis of demographic factors associated with the presence of chronic pain among U.S. adults.

Demographic variable	Adjusted odds ratio	95% CI	*P*
Age*	1.30	1.28–1.33	<0.001
Sex			
Male	Reference		<0.001
Female	1.14	1.07–1.22	
Education level			<0.001
Less than high school	2.89	2.52–3.32	
High school graduate	2.06	1.84–2.30	
College or associate degree	1.59	1.42–1.78	
Advanced or professional degree	Reference		
Race			<0.001
Hispanic	0.48	0.43–0.54	
Non-Hispanic White	Reference		
Non-Hispanic Black	0.79	0.71–0.88	
Other	0.55	0.48–0.64	
Insurance status			0.204
Insured	Reference		
Uninsured	1.10	0.95–1.27	
Marital status			<0.001
Married	0.88	0.82–0.95	
Not married	Reference		

* Odds ratio for age reflects increased odds per 10-year increment in age.

Table 3 presents multivariate analysis of demographic factors associated with more severe pain vs mild or moderate pain. Adjusting for other characteristics, female sex was associated with an adjusted odds ratio of 1.41 (1.25–1.59) for severe pain compared with male sex. Lower educational attainment and Hispanic or non-Hispanic Black race were also associated with greater odds of more severe pain. Being married was associated with less severe pain with an adjusted odds ratio of 0.79 (0.69–0.90).

**Table 3:** Multivariate analysis of demographic factors associated more severe pain (vs mild or moderate) among U.S. adults with chronic pain.

Demographic variable	Adjusted odds ratio	95% CI	*P*
Age	1.03	0.99–1.07	0.140
Sex			
Male	Reference		<0.001
Female	1.41	1.25–1.60	
Education level			
Less than high school	2.53	1.96–3.27	<0.001
High school graduate	1.54	1.22–1.95	
College or associate degree	1.21	0.95–1.53	
Advanced or professional degree	Reference		
Race			
Hispanic	1.38	1.10–1.73	<0.001
Non-Hispanic White	Reference		
Non-Hispanic Black	1.67	1.39–2.01	
Other	1.14	0.84–1.56	
Insurance status			
Insured	Reference		0.518
Uninsured	1.09	0.84–1.43	
Marital status			
Married	0.79	0.69–0.90	0.001
Not married	Reference		

The effects of pain on life and work differed across demographic characteristics. Age was associated with an adjusted odds ratio of 1.10 (1.06–1.14) per 10-year increment for pain affecting life or work. Female respondents had greater odds of pain affecting life or work every day than male respondents, and lower educational attainment was associated with greater odds of pain affecting life or work every day. There were no differences across racial groups after accounting for other factors. Being married was associated with lower odds of pain affecting life or work (Table 4).

**Table 4:** Multivariate analysis of demographic factors associated pain affecting life or work most days or everyday (vs never or somedays) among U.S. adults with chronic pain.

Demographic variable	Adjusted odds ratio	95% CI	*P*
Age	1.10	1.06–1.14	<0.001
Sex			
Male	Reference		<0.001
Female	1.26	1.11–1.42	
Education level			
Less than high school	2.14	1.66–2.76	<0.001
High school graduate	1.54	1.23–1.94	
College or associate degree	1.16	0.93–1.46	
Advanced or professional degree	Reference		
Race			
Hispanic	1.07	0.87–1.31	0.906
Non-Hispanic White	Reference		
Non-Hispanic Black	1.04	0.85–1.28	
Other	0.98	0.73–1.30	
Insurance status			
Insured	Reference		0.206
Uninsured	0.84	0.64–1.10	
Marital status			
Married	0.80	0.71–0.91	0.001
Not married	Reference		

Table 5 presents differences in the impacts of pain on family life across demographic groups. Female sex, lower educational attainment, and being married were associated with greater odds of pain affecting family life most days or every day. Age was not associated with pain affecting family life.

**Table 5:** Multivariate analysis of demographic factors associated pain affecting family most days or everyday (vs never or some days) among U.S. adults with chronic pain.

Demographic variable	Adjusted odds ratio	95% CI	*P*
Age	1.00	0.96–1.04	0.993
Sex			
Male	Reference		<0.001
Female	1.35	1.15–1.58	
Education level			
Less than high school	2.87	2.06–4.00	<0.001
High school graduate	1.85	1.37–2.49	
College or associate degree	1.48	1.08–2.02	
Advanced or professional degree	Reference		
Race			
Hispanic	1.08	0.83–1.39	0.767
Non-Hispanic White	Reference		
Non-Hispanic Black	1.08	0.86–1.37	
Other	1.15	0.82–1.61	
Insurance status			
Insured	Reference		0.332
Uninsured	0.86	0.63–1.17	
Marital status			
Married	1.20	1.03–1.39	0.017
Not married	Reference		

Among all demographic characteristics studied, only the education level was significantly associated with more ineffective pain management. Age, sex, race, insurance status, and marital status showed no significant associated with ineffectiveness of pain management, after accounting for all other factors (Table 6).

**Table 6:** Multivariate analysis of demographic factors associated with ineffectiveness of pain management (vs somewhat or very effective) among U.S. adults with chronic pain.

Demographic variable	Adjusted odds ratio	95% CI	*P*
Age	0.96	0.91–1.00	0.071
Sex			
Male	Reference		0.875
Female	1.01	0.86–1.20	
Education level			
Less than high school	2.17	1.57–3.01	<0.001
High school graduate	1.49	1.11–1.98	
College or associate degree	1.23	0.92–1.64	
Advanced or professional degree	Reference		
Race			
Hispanic	1.04	0.79–1.37	0.842
Non-Hispanic White	Reference		
Non-Hispanic Black	0.92	0.71–1.19	
Other	1.09	0.76–1.56	
Insurance status			
Insured	Reference		0.724
Uninsured	1.06	0.76–1.49	
Marital status			
Married	0.94	0.80–1.11	0.486
Not married	Reference		

## 4. Discussion

In this investigation of a household-based, nationally representative survey, we find considerable variation in the distribution of chronic pain by demographic factors. Because the NHIS is a household-based survey, the current data allow for a robust national estimate of the relationship between these factors and chronic pain without relying on a treatment-seeking or referral population. Overall, chronic pain was more common among women, respondents with lower educational attainment, non-Hispanic White, insured, and not married respondents. After controlling for other socioeconomic variables in a multivariate analysis, age, female sex, and lower educational attainment were independently associated with greater likelihood of having chronic pain, whereas non-White race and being married were associated with lower likelihood of having chronic pain. These findings emphasize the importance of including demographic factors in the global assessment of chronic pain.

Our study demonstrates disparities across racial and ethnic groups in the experience of chronic pain. Compared with non-Hispanic White respondents, Hispanic, non-Hispanic Black, and other race were independently associated with lower odds of having chronic pain. This finding is in contrast with several other chronic conditions, including hypertension and diabetes.^6,9^ Despite lower likelihood of having chronic pain, these respondents were significantly more likely to report severe pain vs mild to moderate pain. The reasons for this difference are unclear, but there are several possible explanations. Access to care has been reliably shown to be lower among non-White American adults because uninsurance rates remain higher among these populations.^11^ It is possible that Hispanic and non-Hispanic Black adults who develop chronic pain are less likely to receive high-quality pain treatment, resulting in higher intensity pain among these patients. However, our data find no evidence of disparities across racial groups in the perceived effectiveness of pain management, and after controlling for other demographic factors of health, insurance was not found to be an independently associated risk factor for more poorly managed pain. There may be racial or ethnic differences in the type of pain syndromes or conditions experienced by different groups, although our data were not able to assess this possibility.

Multiple previous studies have found disparities in chronic conditions across socioeconomic strata.^20^ A large number of chronic diseases, including neurologic diseases, diabetes, and osteoarthritis, have been consistently shown to occur significantly more frequently among patients with lower socioeconomic status.^4^ Individuals with lower levels of socioeconomic status experience greater degrees of stress, increasing their risk of chronic health conditions.^2^ In addition, lower socioeconomic status is associated with fewer resources for managing stress, which may potentiate the increased vulnerability to poorer outcomes experienced by individuals with lower socioeconomic status.^1^ Our study finds lower educational attainment is an independent risk factor for both the development of chronic pain as well as increased pain severity among adults with chronic pain. Individuals with the lowest level of education in our sample were similarly found to have the highest odds of pain affecting life, work, and family most days. Previous research has found increasing socioeconomic disparities in pain prevalence, with greater increases among those with lower incomes and lower educational attainment.^24^ Our study confirms these findings persist through the most contemporaneous data available. Although being at higher risk for more impactful chronic pain, individuals with lower educational attainment also were more likely to report ineffective pain management. It is possible these individuals are less likely to receive comprehensive and multimodal pain treatment that is associated with improved functional outcomes.^22^ Alternatively, the cross-sectional design of our study does not preclude the possibility that greater intensity chronic pain may hinder educational attainment. Additional research is needed into this topic.

We did not find a significant difference in perceived effectiveness of pain management across racial groups after controlling for other demographic factors associated with health. This finding is particularly interesting in light of recent emphasis on racial and ethnic disparities in pain treatment.^13^ Studies in emergency departments have demonstrated differences in the treatment of pain across race and ethnicity, with non-White patients being less likely to receive opioids for painful conditions.^15,16^ Studies across health care settings have found similarly lower likelihood of receiving opioids among non-White patients, while studies of long-term nursing care facility residents with chronic pain have found lower probability of receiving any analgesic among Black and Asian residents when compared with White residents.^8,12^ Black patients have also been shown to be less likely than White patients to receive interventional pain therapies in small studies.^17^ Despite these differences in care, similar self-reported effectiveness was found in our study across racial groups.

The social and occupational impacts of chronic pain are considerable, and there were robust differences across levels of educational attainment in the impacts of chronic pain. Controlling for other demographic factors affecting health, we found more than 2-fold greater odds of pain affecting life or work most days among respondents with less than high school education when compared with advanced or professional degree holders. This finding is consistent with a prior body of the literature suggesting greater disability associated with chronic back pain among patients with lower educational attainment.^5^ In addition, the prognosis of chronic back pain is likely to be worse among these patients. These findings may be mediated directly by the educational level because patients with less education have been found to show greater levels of catastrophic thinking about pain as well as more passive and more maladaptive coping strategies regarding chronic pain.^19^ Patients with lower educational attainment have also been shown to receive fewer benefits from targeted educational interventions regarding chronic pain than more highly educated patients.^14^ Greater disability from chronic pain among respondents with lower educational attainment may compound the disadvantages these individuals have in the job market, which could lead to cyclical economic ill effects further exacerbating these disparities. Owing to the large-scale survey nature of our data source, we are not able to determine the specific aspects of pain management, such as specific treatments, that should be improved for this population, although these suggest pain interventions targeting the patient population with the lowest educational attainment level could have outsized economic and public health benefits.

### 4.1. Limitations

This study has several important limitations. National Health Interview Survey is a multipurpose health survey that serves as a principal source of information on the health of the civilian, noninstitutionalized population of the United States. An advantage of this data source is that it is sampled at the household level and therefore is not biased toward respondents who seek medical care, improving its generalizability to the general population. In addition, the sampling strategy of NHIS is a strength of the data source because NCHS systematically oversamples Black, Hispanic, and Asian populations from all 50 states and the District of Columbia to develop more precise estimates of minority populations. Given the retrospective nature of the data, our analyses may suffer from potential recall bias, although it is unclear whether these effects would be systematic. In addition, it is possible that NHIS includes a nonresponse bias such that nonresponders to the survey differ from responders in important ways, although it is not possible to determine definitively whether this is the case. National Health Interview Survey is a widely used research instrument that is considered to have strong validity. Finally, inherently as a trade-off for nationally representative data on a large scale, the NHIS lacks granularity to describe the pain treatment strategies adopted by respondents, which may differ across demographic factors, and should be an area for increased research focus going forward.

## 5. Conclusion

This study describes disparities in the prevalence and effects of chronic pain across demographic factors among the U.S. adult population. Using data collected at the population level, we find chronic pain prevalence is greater among female respondents, those with lower educational attainment, and non-Hispanic White, while among those with chronic pain, more significant disruptions in daily life are reported by Hispanic and non-Hispanic Black respondents, suggesting areas for targeted public health improvements. Addressing disparities may more effectively reduce the economic and social burdens of chronic pain in the United States.

## Disclosures

The authors have no conflict of interest to declare.
